# Infarction Distribution Pattern in Acute Stroke May Predict the Extent of Leptomeningeal Collaterals

**DOI:** 10.1371/journal.pone.0137292

**Published:** 2015-09-01

**Authors:** Rajeev Kumar Verma, Jan Gralla, Pascal Pedro Klinger-Gratz, Adrian Schankath, Simon Jung, Pasquale Mordasini, Christoph Zubler, Marcel Arnold, Monika Buehlmann, Matthias F. Lang, Marwan El-Koussy, Kety Hsieh

**Affiliations:** 1 University Institute for Diagnostic and Interventional Neuroradiology, Inselspital, University of Bern, Bern, Switzerland; 2 Institute of Radiology, Tiefenau Hospital, Spital-Netz Bern, Bern, Switzerland; 3 Department of Neurology, Inselspital, University of Bern, Bern, Switzerland; University Medical Center (UMC) Utrecht, NETHERLANDS

## Abstract

**Objective:**

The aim of this study was to evaluate whether the distribution pattern of early ischemic changes in the initial MRI allows a practical method for estimating leptomeningeal collateralization in acute ischemic stroke (AIS).

**Methods:**

Seventy-four patients with AIS underwent MRI followed by conventional angiogram and mechanical thrombectomy. Diffusion restriction in Diffusion weighted imaging (DWI) and correlated T2-hyperintensity of the infarct were retrospectively analyzed and subdivided in accordance with Alberta Stroke Program Early CT score (ASPECTS). Patients were angiographically graded in collateralization groups according to the method of Higashida, and dichotomized in 2 groups: 29 subjects with collateralization grade 3 or 4 (well-collateralized group) and 45 subjects with grade 1 or 2 (poorly-collateralized group). Individual ASPECTS areas were compared among the groups.

**Results:**

Means for overall DWI-ASPECTS were 6.34 vs. 4.51 (well vs. poorly collateralized groups respectively), and for T2-ASPECTS 9.34 vs 8.96. A significant difference between groups was found for DWI-ASPECTS (p<0.001), but not for T2-ASPECTS (p = 0.088). Regarding the individual areas, only insula, M1-M4 and M6 showed significantly fewer infarctions in the well-collateralized group (p-values <0.001 to 0.015). 89% of patients in the well-collateralized group showed 0–2 infarctions in these six areas (44.8% with 0 infarctions), while 59.9% patients of the poor-collateralized group showed 3–6 infarctions.

**Conclusion:**

Patients with poor leptomeningeal collateralization show more infarcts on the initial MRI, particularly in the ASPECTS areas M1 to M4, M6 and insula. Therefore DWI abnormalities in these areas may be a surrogate marker for poor leptomeningeal collaterals and may be useful for estimation of the collateral status in routine clinical evaluation.

## Introduction

Several studies have investigated the role of pial collaterals in patients with acute ischemic stroke (AIS). Good collateralization has a protective effect on the penumbra, which might be sustained until recanalization is achieved, since patients with good collaterals have fewer regions of severely hypoperfused tissue compared to those with poor collaterals [1]. The relationship between the collateralization grade and the predictability of infarct evolution was a main study focus in recent years [1–5]. In a few studies a better collateralization grade was associated with better recanalization, reperfusion, and subsequently better clinical outcome [6–9].

More recently, collateralization grading systems based not only on conventional angiograms, but also on CT and MR, were used in several studies [10–15]. In a retrospective CT-based volumetric study Christoforidis et al [16] reported that infarct volume and clinical severity at discharge were lower for patients with better pial collateral status, regardless whether recanalization was complete or partial. Other trials reported correlations between collateralization and infarct volume, perfusion characteristics, and/or extent of prominent cortical veins on susceptibility weighted imaging (SWI) [15,17,18].

Collateralization extent from the anterior and posterior territories to the middle cerebral artery (MCA) territory differs regionally, being greater in the superficial and lesser in the deeper parts of the cerebrum because the main arteries run through the subarachnoid space and their branches penetrate from the outside inwards until the “watershed” of the two territories. We hypothesized that this fact might be useful for estimation of the collateralization grade in initial MRI. The study purpose was to elucidate the relationship between the pretreatment infarction distribution pattern in diffusion weighted imaging (DWI) and the collateralization grade in AIS patients. Furthermore, T2-weighted hyperintense infarcted areas were assessed, since they reflect the stage of vasogenic edema.

The aim of this study was to assess a possible association between ischemic changes in DWI and T2-weighted (T2w) images in the vascular territory of the MCA and the leptomeningeal collateralization grade. Secondarily, we analyzed whether a particular lesion distribution, according to ASPECTS, may be a valuable predictor for good collateralization. Although a few recently published studies showed significant correlation between ASPECTS score and collateralization grade [19, 20], these investigations evaluated ASPECTS in total. To date, evaluation of the anatomical lesion patterns involving individual ASPECTS areas has not been assessed.

## Material and Methods

### Patients

Patients with acute stroke admitted to our stroke center are continuously recorded in the Bernese Stroke Registry. For this retrospective analysis we included consecutive patients who fulfilled the following criteria: (1) diagnosis of AIS established with MRI including DWI and T2w images, (2) presence of complete occlusion of the M1-segment of the MCA, and (3) digital subtraction angiography (DSA) for grading of leptomeningeal collaterals. Patients were excluded if: (1) image quality was poor, e.g. due to motion artifacts, (2) M1-segment was only partially occluded, (3) internal carotid artery was additionally occluded, or (4) time between stroke onset and MRI scan could not be determined, e.g. in “wake up”-stroke subjects.

The initial National Institutes of Health Stroke Scale (NIHSS) scores and time between stroke onset and MRI acquisition were documented.

The study was performed in accordance with the ethical guidelines and permission of the institutional review board.

### Data acquisition

Imaging studies were performed in 1.5T and 3T scanners (Magnetom Avanto and Magnetom Verio respectively; Siemens Medical Solution, Erlangen, Germany) equipped with a 12-channel coil. Standard stroke MRI protocol was performed, which included diffusion-weighted imaging (DWI), T2w imaging, time-of-flight magnetic resonance angiography (TOF-MRA), susceptibility weighted imaging (SWI), perfusion imaging, first-pass gadolinium-enhanced MRA (GE-MRA) of the cervical and intracranial arteries, and T1-weighted (T1w) post-contrast imaging. Parameters of the 1.5T scanner for DWI were: voxel 1.2x1.2x5mm, slices 19, FOV 230x230, slice thickness 5mm, TR3000, TE89, matrix 192x192, averages 4; and for T2w images: slices 24, FOV 230x230, slice thickness 5mm, TR4000, TE99, average 1, matrix 256x235, flip angle 150. Parameters of 3T scanner for DWI were: voxel 1.8x1.8x5, slices 19, FOV 230x230, slice thickness 4mm, TR3500, TE 89, matrix 128x128, averages 4; T2w image parameters: slices 24, FOV 220x192, slice thickness 5mm, TR3760, TE85, average 1, matrix 512x307, flip angle 150. Acquisition time in total: 17 min 58 s.

DSA was performed using a biplane, high-resolution angiographic system (Axiom Artis zee; Siemens, Erlangen, Germany).

### Data analysis

All images were evaluated using our picture archiving and communication system. The collaterals were assessed according to the Higashida Collateral Flow Grading System [21]. This grading system subdivides the collateral flow into five grades, from grade 0 (no collaterals visible on the ischemic side) to grade 5 (complete and rapid collateral blood flow to the vascular bed in the entire ischemic territory by retrograde perfusion). The assessment was performed independently by two interventional neuroradiologists (C.Z. and K.H.), who were blinded to patient history, except for the information of a complete thromboembolic M1-segment occlusion. In case of disagreements a consensus decision was made. According to the grading, the patients were dichotomized into good collaterals (ASPECTS grade 3–4) or poor collaterals (ASPECTS grade 0–2).

ASPECTS, a 10-point semiquantitative CT scoring system, was used to assess the pattern of signal intensity abnormalities in DWI (DWI-ASPECTS) and T2w (T2-ASPECTS) images [22,23]. This score divides the MCA territory into 10 areas of interest (lentiform nucleus, caudate nucleus, internal capsule, and 7 cortical /superficial areas: M1 to M6, and the insula). Therefore, ASPECTS is a topographic scoring system applying a quantitative approach that does not ask physicians to estimate volumes from two-dimensional images. Two diagnostic neuroradiologists (R.K.V. and A.S.), who were blinded to patient history and the collateralization grade, evaluated ASPECTS independently with regard to DRA and hyperintense T2w signal alterations. The T2w signal alterations were only considered valid if they distinctly corresponded with the DRA.

Overall DWI- and T2-ASPECTS were analyzed. Further, all 10 ASPECTS areas were investigated individually. DWI and T2w abnormalities were noted for each area. Results of individual ASPECTS areas were compared between the two groups. Clinical outcomes were assessed at 90 days using the modified Rankin Scale.

### Statistical analysis

Statistical analysis was performed with a commercial software (IBM SPSS Version 20, Armonk, NY).

Comparisons of overall ASPECTS could not be performed in the same manner. For T2-ASPECTS a t-test could not be used because the sum of T2-ASPECTS had a skewed distribution. Since variances for both groups in T2-ASPECTS were about the same, a Mann-Whitney U-test was applied. For DWI-ASPECTS however, the sum distribution was quite symmetrical, but the variances were different. Therefore, a t-test with Welch-correction was used.

Comparisons of ASPECTS of individual areas between the good collateralization and poor collateralization groups were performed by using the Fisher exact test (two-tailed). ASPECTS of the two groups were compared in terms of diffusion restriction and T2-hyperintense infarction demarcation.

### Results all patients (good and poor collateralization groups)

Seventy-four subjects (35 women, 39 men; median age 66.6 years, range 24–86) fulfilled the inclusion criteria. The collateralization in 15 subjects was classified as grade 4, 14 as grade 3, 18 as grade 2, and 27 as grade 1. None of the patients had a collateralization grade 0. Due to the relatively small sample, patients were dichotomized into a “good collateralization” group (n = 29) and a “poor collateralization” group (n = 45). The AIS patients underwent MRI 59–420 minutes after symptom onset (mean 163 minutes). None of these baseline characteristics (e.g. time between symptom onset and MRI scan, age, gender) differed significantly.

All but two patients (97.2%) showed at least one area with diffusion restriction, and 56.7% revealed additional T2 hyperintensity. Mean DWI-ASPECTS and T2-ASPECTS were 5.24 and 9.10 respectively. 18.4% of individual DWI lesions were demarcated in T2-weighted imaging. The inter-rater agreement was 65.51% for T2-ASPECTS and 66.22% for DWI-ASPECTS before consensus reading. The modified Rankin Scale could not be assessed in 3 of 74 (2 with poor and one with good collateralization) due to transfer to another hospital. A summary of patients’ baseline characteristics and the imaging findings are shown in Table 1.

**Table 1:** Overview of patients’ baseline characteristics, and imaging findings in all patients, separated by good vs. poor leptomeningeal collateralization.

	All patients	Good coll. group	Poor coll. group
Number of Patients	74	29	45
Female gender: n/total (%)	35/74 (47.3%)	14/29 (48.3%)	21/45 (46.7%)
Age: mean years	66.6	67.9	65.8
Mean time, symptom onset to MRI scan(min)	163.0	175.1	154.9
Mean NIHSS	14.4	12.1	16.0
Mean modified Rankin Scale (mRS) 90 days post event	2.2	1.6	2.7
Mean DWI-ASPECTS	5.24	6.34	4.51
Mean T2-ASPECTS	9.10	9.34	9.36
Patients with DWI lesions / T2w lesions (%)	97.3 / 56.7	93.1 / 41.3	100.0 / 64.0
Diffusion restricted lesions with T2 hyperintense demarcation (%)	18.4	17.9	18.9

ASPECTS = Alberta Stroke Program Early CT score; DWI = Diffusion weighted imaging; NIHSS = National Institutes of Health Stroke Scale.

### Comparison of good vs poor collateralization groups

In the good and poor collateralization groups MRI acquisition was done within comparable times after stroke onset (175.1 and 154.9 min respectively). NIHSS scores were 12.1 and 16.0, respectively. Mean DWI-ASPECTS was higher in the good collateralization group (6.34) compared to the poor collateralization group (4.51). Mean T2-ASPECTS did not substantially differ between the good collateralization (9.34) and the poor collateralization group (8.95). A significant difference between groups was found for overall DWI-ASPECTS (p<0.001).

### Patterns of DWI- and T2-ASPECTS

**Fig 1:**
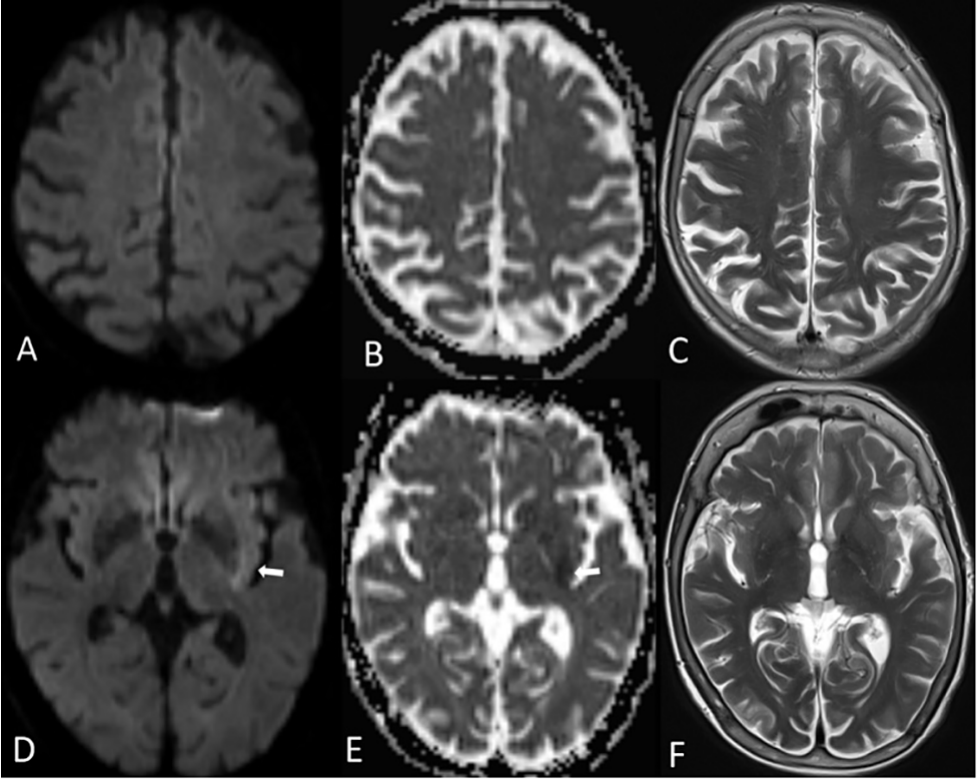
Typical pattern of a well-collateralized patient. 78y old female with left-sided M1-occlusion and a very good pial collateralization (Higashida grade 4); two transverse sections from the acute MRI: DWI (A,D), ADC maps (B,E) and T2w images (C,F). Diffusion restriction was seen in the dorsal part of the lentiform nucleus and the adjacent insula (white arrows in D, E).

**Fig 2:**
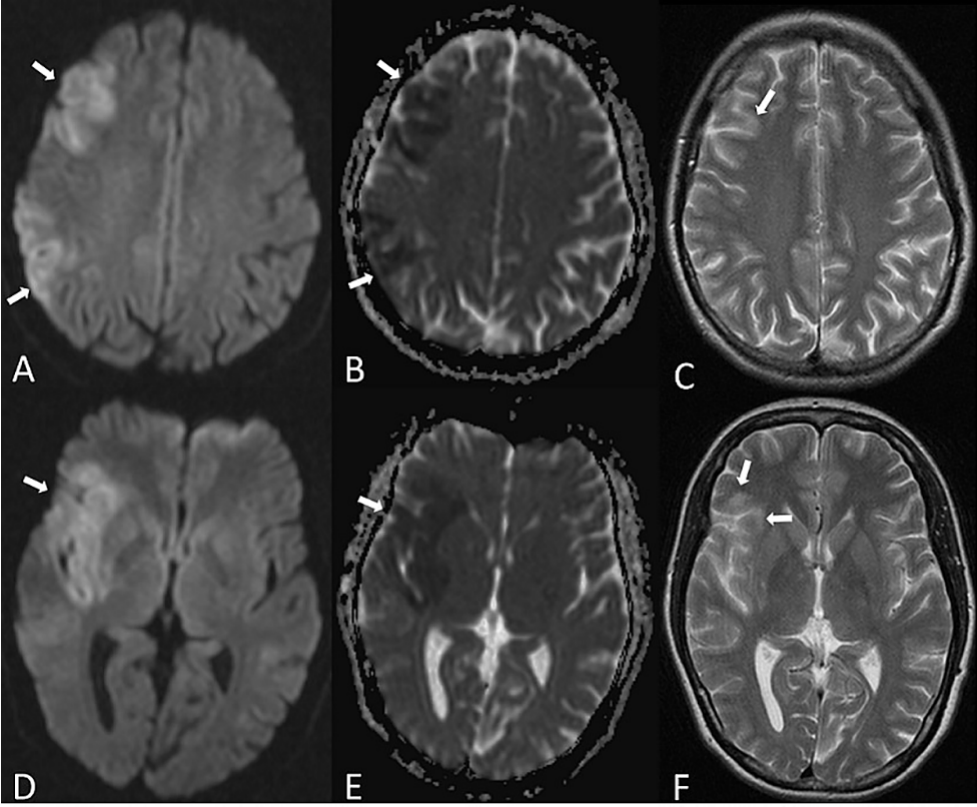
Typical pattern of a poorly-collateralized patient. 39y old female with right-sided M1-occlusion and a poor collateralization grade (Higashida grade 1); DWI (A,D), ADC maps (B,E) and T2w images (C,F). Diffusion restriction (A, B, D and E) is detected in the lentiform nucleus, insula, M1, M2, M4, M5 and M6 according to ASPECTS.

**Fig 3:**
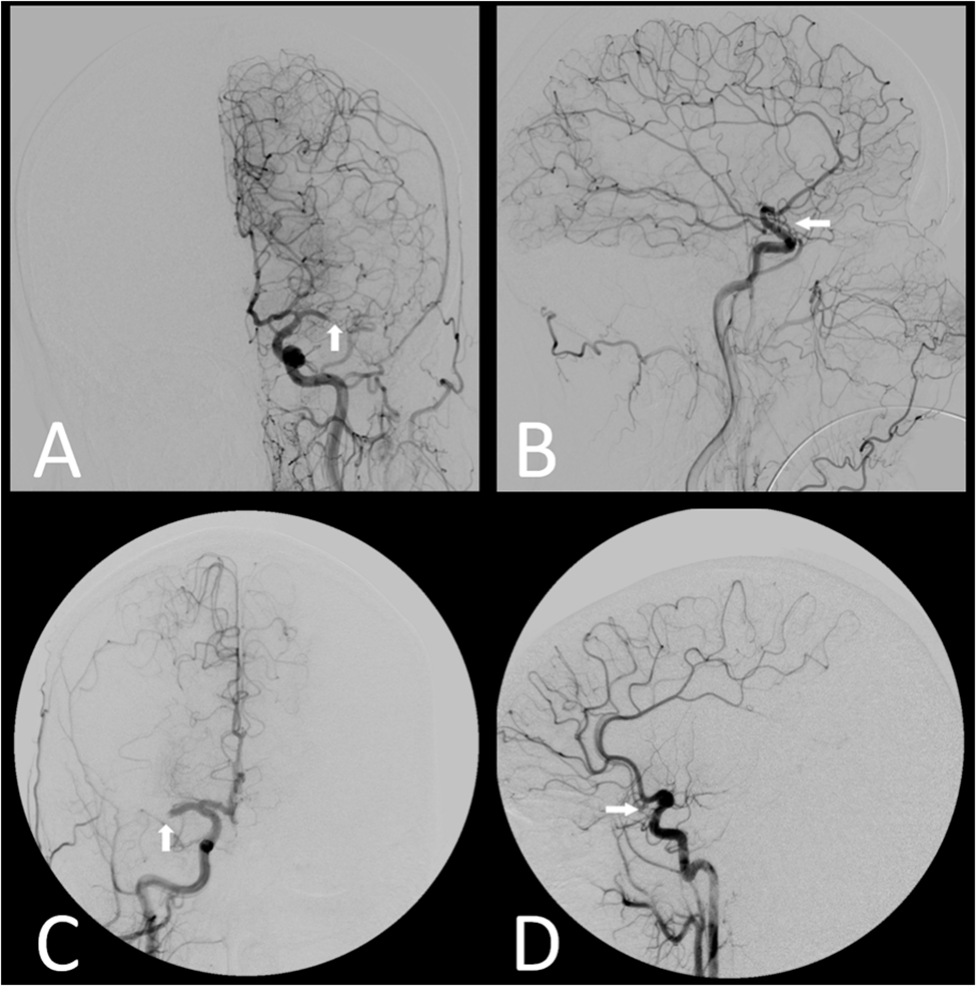
Conventional angiography findings of the patients shown in Fig 1 and Fig 2. Angiography images A and B correspond to the 78y old female patient with good collateralization (Higashida grade 4; Fig 1), while images C and D correspond to the 39y old female with poor collateralization (Higashida grade 1, Fig 2). Both subjects show a M1-segment occlusion of the middle cerebral artery (see arrows).

The DWI-ASPECTS analysis revealed that the following areas were significantly less affected in the good collateralization group than the poorly collateralized group: insula (p = 0.006), M1 (p = 0.011), M2 (p = 0.002), M3 (p = 0.005), M4 (p<0.001) and M6 (p = 0.015). The other areas were evenly affected in the two groups. Most commonly affected areas in DWI-ASPECTS in the whole collective were the lentiform nucleus, caudate, internal capsule, insula and M5 area (Table 2). The remaining 5 areas (M1 to M4, M6) showed fewer diffusion restrictions, especially in the good collateralization group. The only significant difference in T2-ASPECTS between groups was found in the insula (p = 0.02). Median of T2-ASPECTS was 9 for the poorly collateralized group and 10 for the well-collateralized group. Median of DWI-ASPECTS was 4 for the poorly collateralized group and 6 for the well-collateralized group. For individual patient examples see Figs 1–3.

**Table 2:** Patterns of DWI- and T2-ASPECTS.

	Diffusion restriction			T2 hyperintensity		
ASPECTS area	Good coll. group (%**)	Poor coll. group (%**)	*p-values*	Good coll. group (%**)	Poor coll. group (%**)	*p-values*
C	65.5	64.4	*p = 1*.*0*	17.2	8.9	*p = 1*.*0*
I	48.3	80	*p = 0*.*006* *	13.8	40	*p = 0*.*02* *
IC	58.6	57.8	*p = 1*.*0*	3.4	2.2	*p = 1*.*0*
L	82.8	64.4	*p = 0*.*116*	3.4	3.3	*p = 1*.*0*
M1	13.8	42.2	*p = 0*.*011* *	0	2.2	*p = 1*.*0*
M2	27.6	66.7	*p = 0*.*002* *	3.4	6.7	*p = 1*.*0*
M3	6.8	35.6	*p = 0*.*005* *	0	2.2	*p = 1*.*0*
M4	0	31.1	*p<0*.*001* *	0	4.4	*p = 0*.*517*
M5	51.7	68.9	*p = 0*.*15*	3.4	2.2	*p = 1*.*0*
M6	10.3	37.8	*p = 0*.*015* *	0	2.2	*p = 1*.*0*
Total	36.54	52.09	*p<0*.*001* *	4.43	7.43	*p = 0*.*088*

Abbreviations: C = caudate nucleus, I = insula, IC = internal capsule, L = lentiform nucleus, M1 = anterior MCA cortex, M2 = MCA cortex lateral to insular ribbon, M3 = posterior MCA cortex, M4, M5 and M6 are anterior, lateral and posterior MCA territories, respectively, approximately 2 cm superior to M1, M2 and M3, respectively.

* Significant differences between groups for ASPECTS areas.

** Percentage of patients with ischemic changes on DWI and T2w in the defined ASPECTS areas.

### Distribution of infarctions in M1-M4, M6 and insula

**Fig 4:**
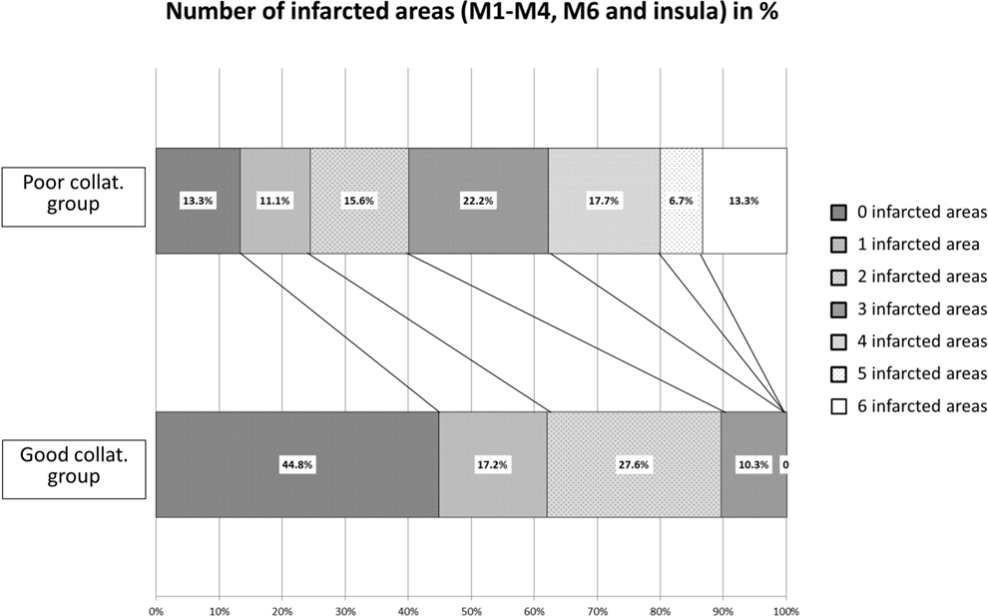
Distribution of infarctions in M1-M4, M6 and insula in patients with good vs. poor collateralization. Almost 90% of well-collateralized patients showed infarctions on DWI in only 0–2 out of the 6 areas, whereas nearly 60% of poorly collateralized patients showed infarctions in 3–6 of the selected areas.

Almost 45% of patients in the good collateralization group but only 13.3% in the poor collateralization group showed no infarctions on DWI in areas M1-M4, M6 and insula. 11.1% vs 17.2% respectively showed one, 27.6% vs 15.6% two, and 10.3% vs 22.2% three infarcted areas. No patient in the well-collateralized group showed more than 3 infarcted areas, whereas in the poorly collateralized group 17.7% had four, 6.7% five, and 13.3% six infarcted areas (see Fig 4).

## Discussion

In our study the initial MRI after AIS onset already showed remarkable differences in infarct demarcation among patients with good or poor collateralization by using ASPECTS classification [22,23]. The good and poor collateralization groups had comparable average times between stroke onset and MRI acquisition, so there was no apparent time bias. In accordance with other studies [12], NIHSS was significantly lower in the well-collateralized group compared to the poorly collateralized group (12.1 vs 16.0, p = 0.0064).

The ***global ASPECT analysis*** showed a significantly higher DWI-ASPECTS in the good collateralization group than in the poor collateralization group (p<0.001), which is in line with earlier reports [1,3,15]. T2-hyperintensity in diffusion restricted areas was found in 56.7% of all patients. Two previous studies had lower rates of T2-hyperintensity (30.3% and 30.0%) detected with FLAIR sequence [24,25]. However, mean time between stroke onset and MRI acquisition was not given in one study, and was 89.7 min in the second, thus much lower than the mean of 162 min in our cohort, which may partially explain the higher rate of T2-hyperintensity in the current study. Further, we included even subtle T2-demarcations in diffusion restricted areas by comparing them with the non-hyperintense symmetric anatomical structures of the opposite side. T2-ASPECTS was not significantly higher in patients with good vs. poor collateralization (p = 0.088). The lack of significance may be due to the small number of T2-hyperintensities. The proportion of T2-hyperintensities in relation to diffusion restricted areas was similar in the two groups (good collateralization group: 17.9%, poor collateralization group: 18.9%). This indicates a comparable T2-hyperintense demarcation evolution in infarcted areas in the “vasogenic edema” phase irrespective of the collateralization. This is not contradictory with earlier reports [24,25] showing a correlation between FLAIR hyperintensities and increased hemorrhagic transformation after thrombolysis, where the extent of T2 demarcation was proportional to the extent of infarction, being more extensive in poorly collateralized subjects.

Regarding the ***individual areas*** of ASPECTS distribution for DWI and T2w, our study revealed differences in infarction extent between groups. Diffusion restriction was detected significantly less often in the insula, M1 to M4, and M6 in well-collateralized subjects (p-values between <0.001 and 0.015). There were no significant differences in caudate nucleus, internal capsule, lentiform nucleus and M5 area. Diffusion restricted infarctions of the M5 area were mainly adjacent to the body of the caudate nucleus, a territory supplied by the lenticulostriate arteries that is deeply penetrated by the MCA. This might explain the comparable infarction rate in the two groups, since a better protective effect of collaterals might be expected due to the peripheral location of the M5 area, but also because periventricular infarcts in the territory of the long insular arteries may not receive enough flow even in the presence of good leptomeningeal collaterals. Concerning T2w images, only the insular area differed significantly between groups; this can be explained by a high infarction rate in the insula in both groups (48.3% and 80.0%) with a significant difference for diffusion restricted areas (p = 0.006), consequently leading to a significant difference for T2-hyperintense areas.

Overall, these results suggest that the tissue protective role of collaterals is distinctly more powerful in the cortical and subcortical areas M1 to 4, M6 and the insula as compared to deeper structures such as the caudate nucleus, internal capsule, and lentiform nucleus. This could be due to the fact that the majority of the leptomeningeal collaterals from the anterior and posterior cerebral arteries supply cortical peripheral areas, whereas the basal ganglia are predominantly supplied by perforators. Therefore, in patients with poor collaterals the protective effectiveness seems to be lower in the peripheral /cortical areas.

In the areas that showed significantly greater protection (M1-4, M6, insula), 89.7% of the well-collateralized group showed only 0 to 2 infarcted areas (including 44.8% with no infarcted areas). In contrast 59.9% of subjects in the poorly collateralized group showed 3 to 6 infarcted areas (Fig 4). Other methods for collateralization grading with cross sectional imaging have been described. However, these methods need CT- or MR-angiography/-perfusion data, particularly with post-processing imaging, e.g. infarct core volumetry or acquisition of Bayesian arterial-tissue delay maps [10–13,15,17,19]. Knowledge of the collateral status might be helpful, since grade of collateralization influences parenchymal sustainability until recanalization [1] and helps in predicting evolution of infarct extension and functional outcome [4–6,8,9]. Our intention was to find a feasible method for estimating the collateralization status solely using conventional MRI sequences. DWI-ASPECTS within 3 or more of the following areas M1-4, M6 and the insula may predict a poor collateralization status.

In a few recently published studies ASPECTS in AIS concerning collateralization grade and/ or functional outcome was examined [19,20]. Yeo et al. [19] compared methods of scoring collaterals on pre-tissue plasminogen activator brain CT angiography for predicting functional outcomes in acute anterior circulation ischemic stroke. Collaterals were assessed with ASPECTS in pretreatment CT-angiography by contrast opacification in arteries distal to the occlusion. As reported by Liebeskind et al., the extent of collateralization was strongly related to ASPECTS at baseline and correlated with successful recanalization [20]. Similar to our study, these studies by Yeo and et al. and Liebeskind et al. revealed a significant correlation between collateralization grade and overall ASPECTS. Another finding of this study was that good collateralization according to ASPECTS was associated with a better day 90 modified Rankin Scale (p<0.001) [20], which is comparable with our results (p = 0.001). No analysis of the individual ASPECTS areas has been reported. Our aim was to separate the more protected individual ASPECTS areas from the less protected ones in order to more precisely predict the collateralization grade.

### Study limitations

The limitations of our study include the retrospective design and the determination of the infarct distribution using the ASPECTS system, which does not allow quantification in terms of infarct volume of the MCA territory. This may lead to distortions, since an infarcted area would be scored as 1 according to ASPECTS, regardless of the volume. Furthermore, we subdivided subjects into two groups, due to the relatively limited number of patients (n = 74). A more detailed analysis in a larger population might be helpful, particularly if it included a more detailed classification of collateralization grades.

In conclusion the tissue protective role of good leptomeningeal collateralization in AIS seems to be more pronounced in six superficial cortical areas defined according to ASPECTS (M1 to M4, M6 and the insula). Further studies will be necessary to verify our findings. Inclusion of a modified ASPECTS with evaluation of only these six cortical areas might be a valuable tool for estimation of the collateral status in routine clinical practice.
